# The neuroprotective potential of mesenchymal stem cells from bone marrow and human exfoliated deciduous teeth in a murine model of demyelination

**DOI:** 10.1371/journal.pone.0293908

**Published:** 2023-11-09

**Authors:** Torbjørn Kråkenes, Stig Wergeland, Niyaz Al-Sharabi, Samih Mohamed-Ahmed, Siren Fromreide, Daniela-Elana Costea, Kamal Mustafa, Lars Bø, Christopher Elnan Kvistad

**Affiliations:** 1 Neuro-SysMed, Department of Neurology, Haukeland University Hospital, Bergen, Norway; 2 Department of Clinical Medicine, Faculty of Medicine, University of Bergen, Bergen, Norway; 3 Tissue Engineering Group, Center of Translational Oral Research (TOR), Department of Clinical Dentistry, University of Bergen, Bergen, Norway; 4 Center for Cancer Biomarkers CCBIO and Gades Laboratory for Pathology, Department of Clinical Medicine, Faculty of Medicine, University of Bergen, Bergen, Norway; 5 Department of Pathology, Haukeland University Hospital, Bergen, Norway; University of Texas at San Antonio, UNITED STATES

## Abstract

**Introduction:**

Multiple sclerosis (MS) is characterized by chronic inflammation, demyelination, and axonal degeneration within the central nervous system (CNS), for which there is no current treatment available with the ability to promote neuroprotection or remyelination. Some aspects of the progressive form of MS are displayed in the murine cuprizone model, where demyelination is induced by the innate immune system without major involvement of the adaptive immune system. Mesenchymal stem cells (MSCs) are multipotent cells with immunomodulatory and neuroprotective potential. In this study, we aimed to assess the neuroprotective potential of MSCs from bone marrow (BM-MSCs) and stem cells from human exfoliated deciduous teeth (SHED) in the cuprizone model.

**Methods:**

Human BM-MSCs and SHED were isolated and characterized. Nine-week-old female C57BL/6 mice were randomized to receive either human BM-MSCs, human SHED or saline intraperitoneally. Treatments were administered on day -1, 14 and 21. Outcomes included levels of local demyelination and inflammation, and were assessed with immunohistochemistry and histology.

**Results:**

BM-MSCs were associated with increased myelin content and reduced microglial activation whereas mice treated with SHED showed reduced microglial and astroglial activation. There were no differences between treatment groups in numbers of mature oligodendrocytes or axonal injury. MSCs were identified in the demyelinated corpus callosum in 40% of the cuprizone mice in both the BM-MSC and SHED group.

**Conclusion:**

Our results suggest a neuroprotective effect of MSCs in a toxic MS model, with demyelination mediated by the innate immune system.

## Introduction

Multiple sclerosis (MS) is an immune-mediated disease of the central nervous system (CNS) characterized by inflammation, multifocal demyelination, and neuronal degeneration. MS is the most common non-traumatic cause of disability in young adults and the incidence has increased over the past decades [1]. Traditionally, MS has been considered a disease triggered by T cell-mediated autoimmune events (outside-in) due to a largely unknown cause, with peripheral immune cells invading the blood-brain barrier causing inflammation and secondary axonal degeneration [2]. The highly prevalent oligoclonal bands in the cerebrospinal fluid and beneficial effect of anti-CD20 therapies point at a central role also for the B cells in the pathogenic cascade [3]. Recently, an alternative view has suggested that the “real MS” may be driven by a primary smouldering process within the CNS with the activation of the immune system representing an epiphenomenon to the primary neuroaxonal loss (inside-out) [4]. This view may explain why some patients with MS experience progression of disease in the absence of new inflammatory disease activity.

Remyelination, which may restore saltatory axon potential conduction and prevent axonal degeneration, frequently fails in patients with MS. As the disease progresses, lack of myelin and neuronal degeneration results in neurological deficits and long-term disability [5]. In recent years, several pharmacological agents have shown beneficial effects by modulating the adaptive immune system and thereby reducing relapse frequency for patients with relapsing-remitting MS and active progressive MS [6]. However, these agents neither modulate the innate immune system nor necessarily stop disease worsening. All agents have potential serious adverse effects and represent life-long therapies. Also, there is no current treatment available with the ability to directly promote neuroprotection or remyelination.

Mesenchymal stem cells (MSCs) exhibit self-renewal potential and have multipotent properties [7]. They can easily be obtained from the bone marrow (BM) and expanded *ex vivo*. MSCs may also be derived from other types of tissues, including dental tissue. Stem cells from human exfoliated deciduous teeth (SHED) reside within the perivascular niche of the dental pulp and are considered a promising source of stem cells due to their stemness and ability to differentiate into other cell lines, such as neurons and oligodendrocytes [8]. The use of autologous or allogeneic MSCs do not evoke the ethical concerns of other stem cell therapies, such as those based on embryonal or fetal stem cells. There is also no need for genetic manipulation, as for induced pluripotent stem cells.

Previous studies have shown that MSCs may have neuroprotective properties [9]. MSCs can migrate towards sites of injury [10], where they are able to adapt to the local environment and promote protection and regeneration of myelin and neurons, thus leading to improved functional outcomes in models of CNS diseases. [11–13] This effect is likely mediated through different mechanisms, such as the paracrine stimulation of endogenous progenitor cells and stem cells through their secretome [14], mitochondria donation [15], immunomodulation [16] and transdifferentiation in neural and oligodendroglial direction [17].

MS occurs exclusively in humans, but different aspects of the disease may be mimicked in disease models. The two most common experimental models for MS in rodents are the cuprizone model and the experimental autoimmune encephalomyelitis (EAE) model. The cuprizone model has the advantage of inducing cerebral demyelination via toxicity to oligodendrocytes without major involvement of the adaptive immune system [18]. Adding cuprizone to the mouse chow results in complete demyelination of the corpus callosum after three weeks of exposure. The demyelination is accompanied by focal inflammation with activation of microglia and gliosis by reactive astrocytes. With minor loss of blood brain barrier integrity and minimal infiltration of lymphocytes, the cuprizone model mimics certain aspects of progressive MS. In this study, we aimed to assess the neuroprotective potential of BM-MSCs and SHED in the cuprizone model.

## Material and methods

### Expansion and characterization of BM-MSCs and SHED

Previously isolated BM-MSCs, under ethical approval from the Regional Committee for Medical and Health Research Ethics in Norway (2013/1248 REK Sør-Øst C), were expanded in Minimum Essential Medium Alpha (αMEM, Thermo Fisher, #32561029) supplemented with 5% human platelet lysate (hPL) and 1% antibiotics (penicillin/streptomycin, Sigma-Aldrich, #P4333) and maintained at 37°C in a humidified incubator containing 5% CO_2_. For isolation of SHED, cells were isolated as described previously [19]. Briefly, human deciduous canine teeth, scheduled for routine extraction, were collected from healthy patients aged 12 years under informed, written consent from parents, in accordance with the protocol approved by the Ethical Research Committee at the University of Bergen, Norway (2009/610 REK Vest). Both BM-MSCs and SHED were obtained from healthy donors in 2020. SHED cells were isolated from pulp tissue by enzymatic dissociation, and single-cell suspension was cultured and maintained in αMEM supplemented with 5% hPL and 1% antibiotics (penicillin/streptomycin). Both BM-MSCs and SHED were expanded in vitro for approximately 14 days to passage 2 before injection. According to the minimal criteria proposed by the International Society for Cellular Therapy (ISCT) to define human MSC, BM-MSCs and SHED were characterized for expression of surface antigens CD73, CD90, CD105, as positive markers, and CD34, CD45, and HLA-DR, as negative markers, using flow cytometry (BD Accuri™ C6). Both stem cell sources were differentiated into osteogenic and adipogenic lineages to test multi-lineage differentiation potential, which was tested using Alizarin Red S and Oil Red O respectively, as previously described [20].

### Mice

Nine-week-old female C57BL/6 mice (n = 70; Taconic, Tornbjerg, Denmark) with a mean weight of 20 gram were housed together in GreenLine type II individually ventilated cages (five mice per cage; Scanbur, Karlslunde, Denmark), in standard laboratory conditions (light/dark cycles of 12/12 h, room temperature of 23.9°C, relative humidity of 22.4% and 20 air changes per hour. Maintenance was performed once a week, and the same individuals handled the animals throughout the experimental period. Mice were acclimatized for one week after arrival. Food and tap water were available ad libitum throughout the acclimatization and trial period. After acclimatization, mice were randomized to different treatment groups. Observers assessing outcomes were blinded to treatment allocation. Treatment groups were mixed before housing to reduce bias. The experiments were carried out following the FELASA guidelines (Federation of European Laboratory Animal Science Associations) and were approved by the Norwegian Animal Research Authority (#24378).

### Cuprizone experiment

**Fig 1:**
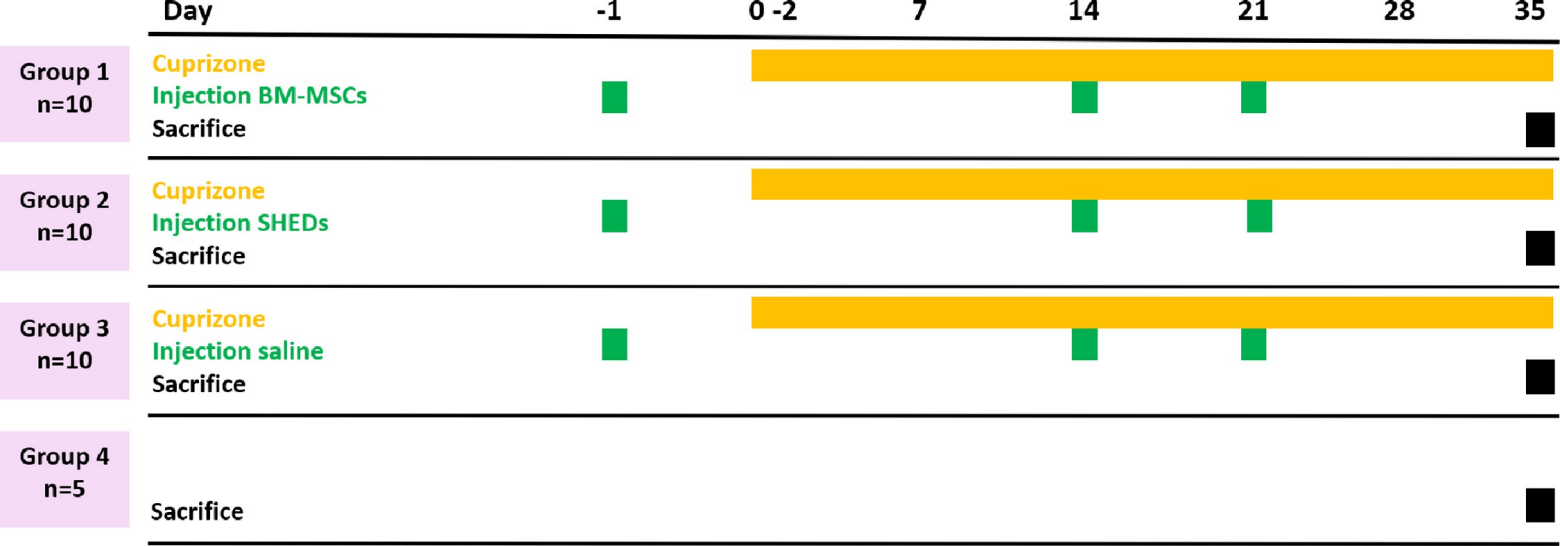
Experimental plan in the cuprizone experiment.

Fig 1 illustrates the experimental design. After acclimatization, cuprizone-mediated demyelination was induced by adding 0.2% cuprizone (bis-cyclohexanone-oxaldihydrazone; Sigma Aldrich, St. Louis, MO, USA) to the ordinary milled mouse chow. Mice were weighed every second day. The cuprizone exposure was continued until time of euthanasia (day 35). Mice in the control group received ordinary milled mouse chow during the same period.

Mice were randomized into different treatment groups: BM-MSC injection group (n = 10), SHED injection group (n = 10), saline injection group (n = 10) and healthy control group (n = 5). Mice in BM-MSC and SHED groups received one million stem cells dissolved in 0.2 ml saline via intraperitoneal injections at day -1, 14 and 21 whereas the mice in the saline group received the same amount of saline through the same administration route. The healthy control group did not receive any injections.

### Histopathology

Animals were anesthetized by isoflurane inhalation, followed by cardiac puncture and perfusion with 4% paraformaldehyde (PFA) through the left cardiac ventricle. Brains were post-fixed for at least 7 days in 4% PFA before they were embedded in paraffin and cut in 5 μm sections ±1 mm from the bregma.

### Immunhistochemistry

Deparaffinization, rehydration and antigen retrieval of tissue sections were done using the PT Link instrument from Dako (Agilent, Santa Clara, CA, US) according to the manufacturers protocol. Sections were blocked in PBS containing 30% SEA BLOCK (Thermo Fisher, #37527) and 1% Triton X-100 (Sigma-Aldrich, #X100) for 90 minutes. Antibodies were diluted in PBS containing 10% SEA BLOCK and 0.1% Triton X-100 and incubated for 1 h. After each antibody incubation, the sections were washed 3x 5 minutes in PBS. Cover glasses were mounted using ProLong Glass with NucBlue (Thermo Fisher, #P36980). Antibodies used: MAC-3 (BD Biosciences, #550292; 1:100), NEFL (Thermo, #PA1-32240; 1:100), GFAP (Sigma, #HPA056030, 1:500), NOGO-A (Thermo Fisher, #PA5-20367; 1:100), human nuclei (Sigma-Aldrich, #MAB1281, 1:100), Alexa Fluor 647 goat anti-rabbit (Thermo Fisher, #A21245; 1:500) and Alexa Fluor 647 goat anti-rat (Thermo Fisher, A21247; 1:500). Luxol Fast Blue staining, used for myelin staining, was performed by the pathology department at Haukeland University Hospital (Bergen, Norway).

### Microscopy and analyses

Sections stained with antibodies were imaged using the Olympus VS120 S6 slide scanner with an 40x air objective and CY5 excitation and emission filters. The images were analysed using the pixel classification tool in the QuPath 0.4.2 software [21]. Images were thresholded to ameliorate background staining. All sections were analyzed in the midline area of corpus callosum (500.000 μm^2^). Sections stained with luxol fast blue for myelin quantification were scored independently in a blinded manner by two observers (CEK/SW), using light microscopy. Score 0 equals total demyelination and score 3 equals normal myelin staining. Sections stained with MAC-3 (microglia) and GFAP (astrocytes) were scored 0–3 where 0 equals no signal and 3 equals abundant signal. Sections stained with NEFL were analyzed using the average staining intensity in corpus callosum. Oligodendrocytes (NOGO-A) and human cells were counted manually.

### Statistical analyses

For sample size estimation, a systematic search using PubMed, MEDLINE and Web of Science was performed to identify prior trials using SHED in murine EAE or cuprizone models. The search string was “animal or animal model” AND “multiple sclerosis or MS” AND “stem cells from human exfoliated deciduous teeth” or “SHED”. The search was performed in June 2020. One trial was identified using SHED in an EAE model and the EAE clinical scores from this trial was used for sample size estimation [22]. Using a recommended formula for sample size calculation in animal studies for comparison between two groups using quantitative data with alpha of 0.05 and beta of 0.2 (power = 80%), a sample size of 10 animals in each group was calculated [23].

The Shapiro–Wilk test was used to test normal distribution. Students t test was used to analyze parametric data and The Mann–Whitney U test for non‐parametric data. An interrater analysis was performed by calculating the kappa statistic to assess conformity in the rating of myelination in the luxol fast blue staining. All calculations are carried out using STATA 17.0 (Stata Corp, TX, US). Differences were considered significant at p < 0.05.

## Results

### Characterization of human BM-MSCs and SHED

**Fig 2:**
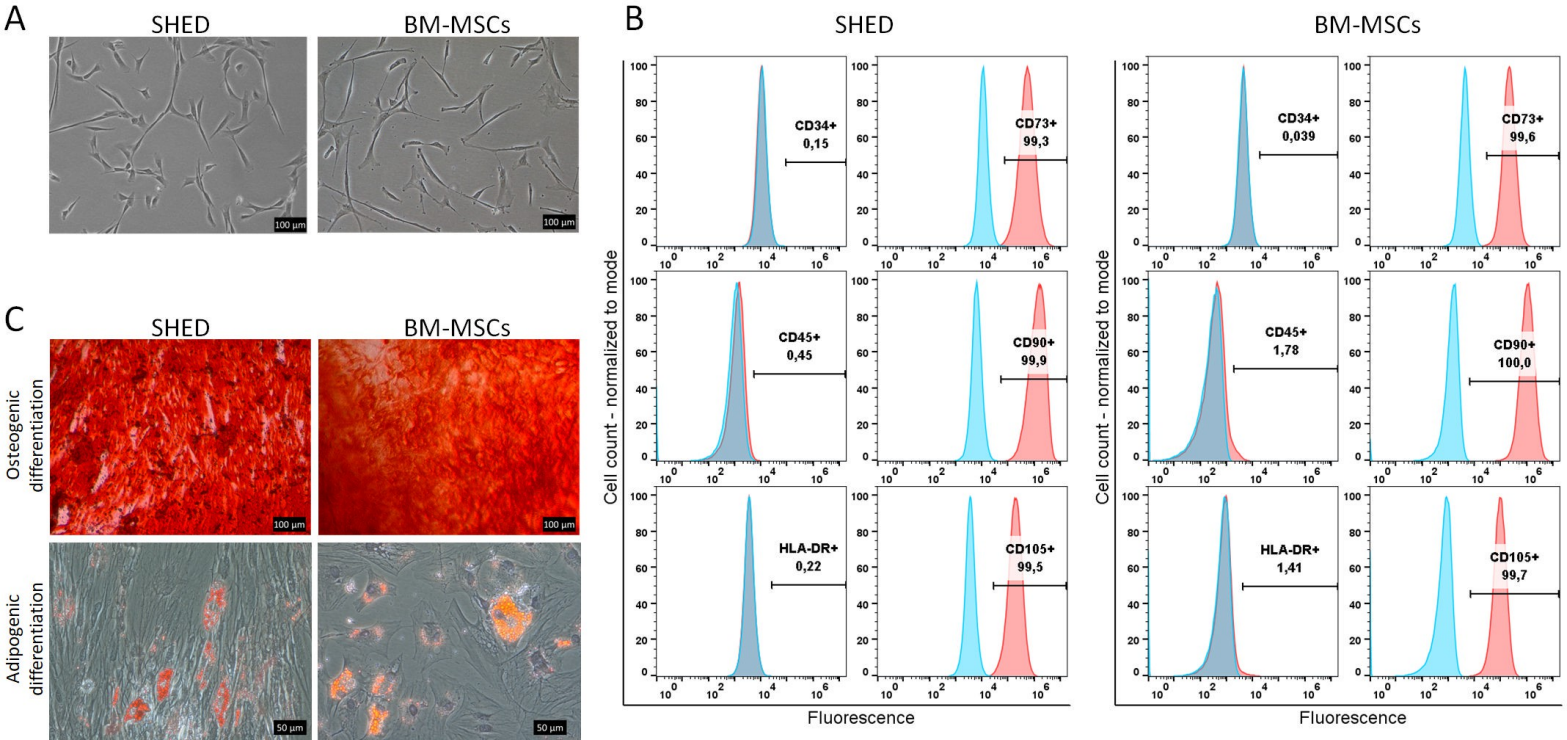
In vitro characterization of BM-MSCs and SHED. **A**) Morphology and proliferation of BM-MSCs and SHED. Cells showed a spindle-like fibrous morphology. **B**) Surface markers of BM-MSCs and SHED using Flow cytometry analyses. The expression of the positive markers, CD73, CD90 and CD105, was present in more than 95% of the cells, while that of the negative markers, CD34, CD45 and HLA-DR, was present in less than 2% of the cells. **C**) Multi-differentiation potential of cultured BM-MSCs and SHED. Osteogenic differentiation for 21 days followed by staining with Alizarin Red S. Adipogenic induction and differentiation for 21 days followed by staining with Oil Red O. Scale bars A-E = 200 μm, F = 50.

As observed by phase-contrast microscopy, BM-MSCs and SHED displayed a consistent, spindle-like fibrous morphology (Fig 2A). The dissimilarity in cellular morphology between the two cell types was negligible. As time progressed, there was a gradual increase in cell number. The flow cytometry analysis showed that all BM-MSCs and SHED highly expressed CD73, CD90 and CD105, with low expression of CD34, CD45 and HLA-DR (Fig 2B). The expression of the positive markers was more than 95%, while that of the negative markers was less than 2%, which meets the minimum standard of the International Society for Cell Therapy (ISCT). Next, we evaluated the osteogenic, and adipogenic differentiation potential of BM-MSCs and SHED using different induction media in vitro (Fig 2C). In the osteogenic differentiation medium, both types of stem cells showed similar solid mineral deposits after 21 days of incubation. Moreover, under Oil Red O, both types of stem cells showed staining for adipogenic differentiation after 14 days of incubation, as evidenced by lipid droplet formation.

### Cuprizone experiment

**Fig 3:**
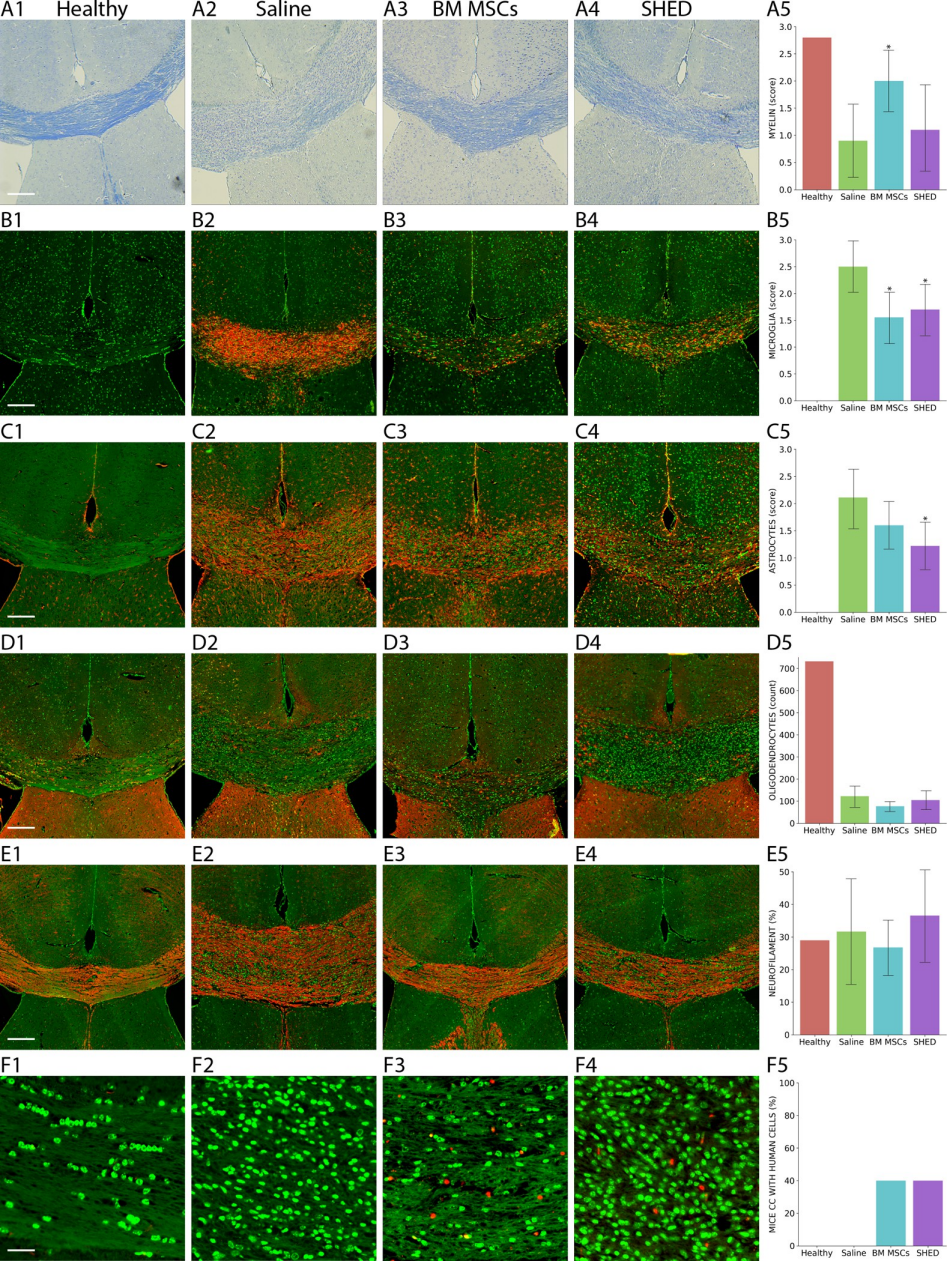
Analysis of corpus callosum in the cuprizone experiment. **A**) The myelin staining (blue) was strongest in the healthy controls (A1). The cuprizone mice that received BM-MSC (A3) had significantly more myelin compared to the saline group (A2). SHED injections (A4) did not give significant changes compared to saline. Statistical data summarized in the graph (A5). **B**) The microglia staining (red; MAC3) was negative in healthy mice (B1). Both in mice receiving BM-MSCs (B3) and SHED (B4), there was significantly less microglia compared to saline (B2). **C**) The astrocyte staining (red; GFAP) showed substantial less activation of astrocytes in healthy mice (C1) compared to the cuprizone mice. In the treatment groups, there was significantly less astrocyte activation in mice that received SHED (C4) compared to saline (C2), while in mice receiving BM-MSCs (C3) there was no significant reduction in astrocyte activation. **D**) In healthy controls (D1), the oligodendrocyte staining (red; NOGO-A) yielded 732 positive cells/cm^2^ on average. In the cuprizone mice, there were substantially less oligodendrocytes, however no differences were found between the treatment groups (D2-D4). **E**) The neurofilament staining (red; NEFL) showed no statistical differences between healthy controls (E1), saline (E2), BM-MSCs (E3) and SHED (E4). **F**) Human cells (red) were found in 4/10 mice in the BM-MSC group (E3) and in 4/10 mice in the SHED group (F4). No human cells were found in healthy controls (F1) or saline-treated mice (F2). Cell nuclei displayed in green (B-F). Scale bars = 200 μm.

In healthy control mice, the myelin staining (luxol; blue) was strong throughout the corpus callosum (Fig 3; A1). The amount of myelin in BM-MSCs mice (Fig 3; A3) was significantly higher than those that received saline (Fig 3; A2). Cuprizone mice that received SHED (Fig 3; A4) had a higher average amount of myelin compared to saline, however, the difference was not significant (Fig 3; A5).

For activated microglia (MAC-3; red), little to no staining was found in healthy controls (Fig 3; B1). In contrast, the corpus callosum of the cuprizone mice receiving saline was filled with activated microglia (Fig 3; B2). The mice receiving both BM-MSCs (Fig 3; B3) and SHED (Fig 3; B4) had significantly less activated microglia compared to the saline group (Fig 3; B5).

The astrocytic marker GFAP (red) showed weak staining in healthy control mice (Fig 3; C1), while there was a profound activation/infiltration of astrocytes in the corpus callosum of cuprizone mice receiving saline (Fig 3; C2) and BM-MSCs (Fig 3; C3). In the mice receiving SHED (Fig 3; C4), there were significantly less activated astrocytes compared to saline (Fig 3; C5).

In healthy control mice, the oligodendrocyte marker NOGO-A (red) showed long rows of oligodendrocytes throughout the corpus callosum (Fig 3; D1), with 732 positive cells/cm^2^ on average. In the cuprizone mice (all treatment groups), the NOGO positive cells were considerably fewer and only a few cell rows were remaining (Fig 3; D2-D4). In these mice the average amount of NOGO positive cells were 103 cells/cm^2^, however, no significant differences were found between the treatment groups (Fig 3; D5).

As for the neuronal filament marker NEFL (red), no significant changes in staining intensity per area were found between the healthy controls (Fig 3; E1) and the cuprizone mice (Fig 3; E2-E4). Furthermore, there was no difference between the amount of NEFL in the treatment groups (Fig 3; E5).

The antibody detecting human cells (red) were positive in 40% of mice in both the BM-MSC group and in the SHED group (Fig 3; F3-F5), staining several cells throughout the corpus callosum. In the healthy controls and in the saline group there were no cells positive for the human marker (Fig 3; F1-F2). There was, however, no correlation between the presence of MCSs and amount of myelin or inflammation with activated microglia in corpus callosum.

## Discussion

In the cuprizone model, where demyelination is induced without major involvement of the adaptive immune system, our study showed increased levels of myelin in mice treated with BM-MSCs compared to those that received saline. This finding suggests a myelin-protective effect for BM-MSCs. However, no such effect was found with SHED, and there was no impact on numbers of mature oligodendrocytes or axonal injury. Inflammation, characterized by microglial activation was lower in both BM-MSCs and SHED, whereas SHED also showed lower astrocyte activation relative to mice treated with saline, suggesting that both BM-MCS and SHED may reduce the innate immune activation within the CNS induced by cuprizone.

Our findings are in opposition to another study, which did not find any positive effects on myelin or inflammation of neither intravenously nor intranasally injected MSCs in a cuprizone model [24]. The study showed that the MSCs were located in the peripheral gray matter in a hematogenic distribution pattern, but not in the demyelinated corpus callosum, indicating that the MSCs did not cross the blood-brain barrier. In contrast, our study identified MSCs in the corpus callosum in 40% of the mice receiving stem cells, both in the BM-MSC and SHED group. There was, however, no correlation between the presence of MCSs and amount of myelin or inflammation in corpus callosum. This may suggest that the presence of MSCs at the time of euthanization is not necessary for a beneficial effect. The time gap of two weeks between last MSC injection and eutanization may also explain why the stem cells were not found in all mice. We cannot rule out that the MSCs in the remaining 60% did not survive this length of time. Furthermore, the cuprizone model do not cause significant damage to the blood brain barrier. This may also account for the incomplete presence of MSCs in the corpus callosum of the treatment groups.

Our findings of a positive effect of BM-MSCs in the cuprizone model are in conformity with another study where murine BM-MSCs were injected intraperitoneally in both cuprizone and EAE mice [25]. The BM-MSCs did not affect the EAE disease course, but higher expression of myelin was found in the corpus callosum of cuprizone mice treated with BM-MSCs, indicating a protective effect on demyelination. The authors discussed whether the beneficial effect on myelin abundance in the cuprizone model could be due to a positive impact of BM-MSCs on survival of oligodendrocyte precursor cells (OPCs) and/or oligodendrocytes during apoptosis. This hypothesis was based on an *in vitro* experiment where factors secreted from BM-MSCs led to increased survival of OPCs [25]. Our study also showed a positive effect of BM-MSCs on expression of myelin in cuprizone mice. We did, however, not observe any differences in numbers of NOGO A-positive cells between treatment groups. As NOGO-A is known to be strongly expressed in mature oligodendrocytes *in vivo*, we cannot rule out that the MSCs had a positive effect on immature OPCs [26].

SHED showed decreased inflammation and astrocytosis in the cuprizone model as compared to saline. To the best of our knowledge, SHED have not been tested in cuprizone mice previously. SHED are derived from the normal dental pulp and originate from the cranial neural crest. These cells exhibit neural stem cell markers and secrete a variety of neurotrophic growth factors that are thought to have great neuroprotective potential [27]. In concert with our findings, other studies have also found a beneficial effect of SHED on microglia in other neurological conditions. In a rodent Alzheimer model, intranasal administration of SHED conditioned medium suppressed inflammatory microglia activation and improved cognitive function [28]. Another study showed that SHED conditioned medium promoted recovery of mice in a spinal cord injury model via modulation of microglia phenotypes and anti-inflammatory activity [29]. Secretome analysis of the SHED conditioned medium identified monocyte chemoattractant protein-1 (MCP-1) and the secreted ectodomain of sialic acid-binding Ig-like lectin-9 (ED-Siglec 9) as major factors in downregulating the inflammatory activity of the microglia. A third study reported suppression of microglia and astrocyte activation in the spinal cord after intravenous administration of SHED and SHED conditioned medium, thereby alleviating allodynia induced by spinal nerve transection [30]. In our study, we did not find any increased myelin abundance or reduced axonal injury after SHED administration, despite the effect on activated microglia and astrocytes. This was partly in contrast to the BM-MSCs, which increased myelin but did not significantly affect astrocyte activation. Studies have shown that astrocytes may contribute to myelination via communication and transfer of lipids to oligodendrocytes [31, 32]. A possible explanation for the lack of myelin in the SHED mice may thus be related to the active role of astrocytes in remyelination.

Our findings may have important implications for patients with MS, especially for patients with progressive MS. The cuprizone model shares many important characteristics of progressive MS, including the progressive clinical worsening with demyelination and activation of innate immune cells in the brain, i.e. the microglia and astrocytes [18]. In contrast to cells of the adaptive immune system, these inflammatory cells are not accessible for the current immunomodulatory treatments used for MS patients. There are so far no means of promoting remyelination or preventing demyelination in patients with progressive disease. Our results indicate that MCSs can migrate to areas in the brain which resemble progressive MS disease, where they reduce inflammation and where the BM-MSCs increased myelin content. However, the analogy between progressive MS and the toxic cuprizone mouse model should be interpreted with caution as both etiology and pathophysiology differ greatly.

There are some limitations of the present study. We used BM-MSCs and SHED from one healthy donor. As these cell populations have been shown to be heterogenous, we cannot rule out that this may have affected the results, also in comparison with other studies as previously mentioned. However, the stemness and cell characteristics of both populations were confirmed prior to the experiment. In addition, we only used a NOGO-A antibody for oligodendrocytes in the cuprizone experiment. NOGO-A is a well-suited marker for mature oligodendrocytes. Ideally, we should also have used a marker for OPCs to elucidate the mechanisms behind the positive effects of BM-MSCs on myelin abundance. Also, behavorial tests, such as the rotarod test, could have given information to the effect of MSCs on motor performance and thereby possibly supporting the histological findings. A strength of the study is the implementation of a blinded, randomized design and use of a well established model for demyelination.

In conclusion, we showed that both BM-MCSs and SHED reduced neuroinflammation in the cuprizone model, whereas BM-MSCs were associated with increased myelin. These findings suggest a neuroprotective effect of BM-MSCs and SHED in a toxic MS model without substantial involvement of the adaptive immune system.

## Supporting information

S1 File (XLSX)
